# Bio-synthesis, purification and structural analysis of Cyclosporine-A produced by *Tolypocladium inflatum* with valorization of agro-industrial wastes

**DOI:** 10.1038/s41598-024-63110-y

**Published:** 2024-05-31

**Authors:** Fereshteh Falah, Ali Samie, Seyed Ali Mortazavi, Abolghasem Danesh, Farideh Tabatabaei Yazdi, Mohammad Ramezani

**Affiliations:** 1 Department of Food Science and Technology, Faculty of Agriculture, Ferdowsi University of Mashhad, Mashhad, Iran; 2 Department of Medicinal Chemistry, School of Pharmacy, Mashhad University of Medical Sciences, Mashhad, Iran; 3 Targeted Drug Delivery Research Center, Pharmaceutical Technology Institute, Mashhad University of Medical Sciences, Mashhad, Iran; 4 Biotechnology Research Center, Pharmaceutical Technology Institute, Mashhad University of Medical Sciences, Mashhad, Iran; 5 Pharmaceutical Research Center, Pharmaceutical Technology Institute, Mashhad University of Medical Sciences, Mashhad, Iran

**Keywords:** Cyclosporine, *Tolypocladium inflatum*, Purification, Chemical structure, Biochemistry, Biotechnology, Microbiology

## Abstract

Cyclosporine A (CyA) holds significant importance as a strategic immunosuppressive drug for organ transplant patients. In this study, we aimed to produce pure and cost-effective Cyclosporine A (CyA) by fermenting a culture medium containing dairy sludge, using *Tolypocladium inflatum* PTCC 5253. Following the fermentation stage, ethyl acetate extraction and fast protein liquid chromatography were employed for sample purification. The initial evaluation of the effectiveness of CyA obtained from these processes was performed through bioassay, wherein the antimicrobial clear zone diameter was found to be larger compared to the sample obtained from the fermentation culture. The concentration of CyA was determined using high-performance liquid chromatography, yielding values of 334 mg/L, 456 mg/L, and 578 mg/L for the fermented, extracted, and purified samples, respectively. Further analysis utilizing liquid chromatography tandem mass spectrometry (LC/MS/MS) confirmed a purity of 91.9% and proper agreement with the standard sample based on the ion intensity of Z/m 1205. To validate the structure of CyA, nuclear magnetic resonance spectroscopy, Fourier-transform infrared (FT-IR), and Raman spectroscopy were employed. X-ray diffraction and differential scanning calorimetry analyses demonstrated that the purified CyA exhibited a crystal structure similar to the standard sample, characterized by two broad peaks at 2θ = 9° and 20°, and comparable glass transition temperatures (57–68 °C for the purified sample; 53–64 °C for the standard sample). Dynamic light scattering analysis confirmed a uniform particle size distribution in both the purified and standard samples. The zeta potentials of the purified and standard samples were determined to be − 25.8 ± 0.16 and − 23.63 ± 0.12 mV, respectively. Our results demonstrate that dairy sludge can serve as a suitable culture medium for the production of (CyA).

## Introduction

Microorganisms are known for their ability to produce metabolites that have a profound effect on human health and nutrition in diverse domains^1^. These metabolites hold immense promise and are actively investigated in fields such as medicine, agriculture, food science, and environmental sustainability. In the realm of medicine, metabolites derived from microorganisms have revolutionized healthcare by giving rise to life-saving drugs. Antibiotics, immunosuppressants, and anticancer agents represent just a few examples of these invaluable contributions. They have played a pivotal role in significantly improving the treatment of infectious diseases, enhancing organ transplantation outcomes, and advancing cancer therapies^2–4^. Indeed, fungi are renowned for their ability to produce a diverse array of secondary metabolites, commonly referred to as fungal natural products. These metabolites have captured significant scientific interest and have been extensively explored. Fungal secondary metabolites exhibit remarkable structural and functional diversity. They play various crucial roles in the natural environment, contributing to the survival and competitive advantage of fungi. Some metabolites serve as defense mechanisms against predators or inhibit the growth of competing microorganisms. Notable examples of these compounds include antibiotics, antifungals, and mycotoxins. Beyond their ecological functions, fungal secondary metabolites have demonstrated immense potential in numerous applications. Many of these metabolites possess valuable pharmacological properties and have been harnessed as sources of drugs and lead compounds for pharmaceutical development^5,6^. Iconic examples encompass the antibiotic penicillin, the immunosuppressant cyclosporine, and the cholesterol-lowering drug lovastatin^7^. Fungal secondary metabolites have a significant effect on various biological activities, despite not being essential for fungal growth. These compounds, although not directly involved in the fundamental processes of growth and development, play crucial roles in numerous biological functions. They contribute to ecological interactions, such as competition and defense mechanisms, and can influence the behavior and physiology of other organisms. Additionally, fungal secondary metabolites exhibit a wide range of bioactive properties, making them highly significant in fields such as medicine, agriculture, and biotechnology. Their diverse biological activities highlight their importance and potential for further exploration and application^3,8,9^. Among the diverse range of fungi, including *T. inflatum*, *Fusarium*, *Aspergillus*, *Trichoderma*, and *Penicillium* species, it is *T. inflatum* that is primarily responsible for the production of CyA^10,11^. In addition to its well-known immunosuppressive function, CyA is widely utilized as an effective and safe medication for the treatment of autoimmune diseases^8,12–17^. CyA is a cyclic polypeptide with the chemical name Cyclo[[(E)-(2S, 3R, 4R)-3-hydroxy 4-methyl-2-(methylamino)-6-octenoyl]-l-2-aminobutyryl *N*-methylglycyl *N*-methyl-l-leucyl-Lvalyl-*N*-methyl-l-leucyl-l-alanyl d-alanyl-*N*-methyl-l-leucyl-*N*-methyl-l-leucyl-Nmethyl-l-valyl]. CyA exhibits several distinct chemical characteristics. Its structure comprises 10 non-polar aliphatic amino acids and one MeBmt amino acid. CyA contains eight methyl group substituents, which contribute to its strong lipophilic properties. Methyl groups are present on the nitrogen atom of certain amino acids within CyA. Additionally, CyA includes an unusual amino acid, (2S, 3R, 4R, 6A)-3-hydroxy-4-methyl-2-methylamino-6-octenoic acid. The amino acid chain of CyA exhibits an asymmetric beta sheet configuration, with three hydrogen bonds remaining in positions 1–6. In the biosynthesis of CyA, four processes are involved in the activation of each amino acid: *N*-methylation, peptide bond formation, and ring formation reactions^18,19^.

With the increasing population and the growing demand for energy resources, various technologies have emerged as solutions to address the issue of resource scarcity. One of these solutions involves utilizing waste from different factories to produce useful and valuable products. Many of these waste sources contain compounds such as carbohydrates, proteins, and vitamins. Therefore, utilizing waste from the food industry, particularly the dairy industry, to produce valuable products like amino acids appears to be a promising approach. This approach can help reduce production costs and minimize food resource waste. Dairy industry waste, known as dairy sludge, is generated during the treatment of dairy wastewater. This waste can serve as a carbon and nitrogen source for microorganisms. Dairy sludge typically represents 0.5–1% of the volume of milk, with a dry matter content of 14–16%. It contains approximately 6–8% nitrogen, 0.25–0.35% fat, 4.7% lactose, and 1.5–3% non-dairy substances^20^. The aim of this study was to produce pure and cost-effective CyA by utilizing dairy sludge as the base culture medium.

## Materials and methods

### Materials and culture media

The MYA (Malt Yeast Extract Agar) and PDA (Potato Dextrose Agar) media utilized in this study were sourced from Merck (Germany). The extraction process involved the use of solvents and reagents obtained from Sigma (Canada). The dairy sludge used in the experiment was obtained from Pegah Khorasan factory and subsequently dried using a spray dryer^20^.

### Microorganisms

*Tolypocladium inflatum* PTCC 5253 and *Aspergillus niger* PTCC 5013 were obtained from the Persian Type Culture Collection.

### Inoculum development

The MYA culture medium was used for the growth of *T. inflatum*, while the PDA culture medium was used for the growth of *A. niger*. The strains were inoculated onto agar slants containing their respective culture media and incubated at a temperature of 27 °C for 14 days for *T. inflatum* and 72 h for *A. niger*^21^.

### Fermentation

To conduct submerged fermentation for CyA production, 200 µL of seed inoculum containing 10^6^ conidia of *T. inflatum* was added to 500 mL Erlenmeyer flasks containing 100 mL of the fermentation medium. The composition of the fermentation medium consisted of 46.47% carbon source, which was a mixture of dairy sludge and glucose in a 1:1 ratio. The nitrogen source comprised 9.56% malt extract and ammonium sulfate in a 1:1 ratio. Additionally, 1.49 mL of trace element solutions (TES) (containing HCl, ZnCl_2_. MnCl_2_ 4H_2_O, H_3_BO_3_, CoCl_2_ 6H_2_O, CuCl_2_ 2H_2_O, NiCl_2_ 6H_2_O, Na_2_MoO_4_ 2H_2_O) was added to the fermentation medium. The medium was adjusted to pH 5.7 and incubated at 27 °C in a shaker incubator (Clim-o-Shake, IRC-17) at a speed of 200× rpm for a period of 10 days. At the 10th day of fermentation, ultrasound treatments (30% total pressure in 5 min, 1 pulse) were applied^22^.

### Extraction

To extract CyA, the culture sample (50 mL) was mixed with ethyl acetate solvent (50 mL) in a 500 mL polypropylene centrifuge bottle (Beckman). The mixture was then placed on a desktop gyratory shaker (GFL 3015) and left overnight for thorough mixing. After the shaking period, the sample was centrifuged at 1500× rpm for 15 min. Following centrifugation, 40 mL of the supernatant was carefully withdrawn from the bottle. The withdrawn supernatant was subjected to evaporation and drying by blowing air at room temperature. Finally, the resulting dried organic sample was dissolved in acetonitrile of LC grade. The quantity of CyA was determined using HPLC^23^.

### Purification of CyA using FPLC with hydrophobic interaction column

As part of the purification process for CyA, column chromatography was performed using the following steps:

Sample application: 0.5 mL of the dissolved sample was carefully applied to the top of a glass column (12 × 600 mm) filled with silica gel 40 powder.

Elution: The sample was eluted isocratically using a mobile phase consisting of a 95:5 (v/v) mixture of ethyl acetate and *n*-hexane.

Fraction collection: A total of 50 fractions, each with a volume of 5 mL, were collected using a model UPC-900 fraction collector.

Analysis: The presence of CyA in the collected fractions was subsequently assessed using the following methods:

Bioassay: A biological assay was conducted to determine the presence of CyA.

Thin layer chromatography (TLC): TLC was performed to visualize the separation of compounds in the fractions.

HPLC: HPLC analysis was carried out to quantify and confirm the presence of CyA^24^.

### Bioassay analysis

To detect the presence of CyA, the diffusion method in agar was employed. A culture of *A. niger* (1 × 10^7^ spores/mL) was prepared and spread on agar plates. The fractions containing CyA (100 µL) was then added to the wells created on the plate's surface. Following an incubation at 25 °C for 3 days, the presence of CyA was assessed by observing the formation of a clear area surrounding the wells. This clear area indicates the inhibition of growth around the wells, which is attributed to the presence of CyA. This method allows for the qualitative determination of CyA in the sample^1,25^.

### Identification of CyA production by TLC

During the TLC analysis, 5 µL of the dissolved sample, along with CyA authentic standard and a CyA purified solution, were carefully applied onto heat-activated silica gel-60 aluminum sheets. These sheets were placed into a TLC tank that had been saturated with a solvent mixture consisting of 95% ethyl acetate and 5% isopropanol (v/v). The chromatography process occurred as the eluent gradually ascended the sheet. Once the eluent reached the top of the sheet, the sheets were carefully removed, dried, and subsequently developed under UV light at a wavelength of 254 nm^26–28^.

#### HPLC analysis

The measurement of CyA at various stages, including post-fermentation, post-extraction, and post-purification, was conducted using HPLC following a method described by Behbahani et al.^29^ with slight modifications. For HPLC analysis, the mobile phase consisted of a mixture of acetonitrile and water in a ratio of 70:30. The flow rate of the mobile phase was maintained at 0.7 mL/min. A 5 μm Lichrospher C18 RP Select B column was used for the chromatographic separation. The column temperature was set to 70 °C, and the working pressure was maintained at 18 kg/cm^2^. During analysis, the HPLC profile was monitored and analyzed at a wavelength of 210 nm. The amount of CyA present in the samples was quantified by comparing the peak area or peak height with the calibration curves generated from CyA standard solutions^30^.

### Investigating the structural and chemical properties of CyA

#### Assessment by LC/MS/MS

The chromatography experiment was carried out employing an Ultra-Fast LC (UFLC) system, consisting of an LC-20AD pump and a SIL-20A autosampler from Shimadzu, Japan. For the separation process, a C18-Supelco column with dimensions of 250 mm × 4.6 mm × 3 μm was utilized. The column temperature was maintained at a constant 75 °C throughout the analysis. Data collection was programmed to capture mass spectra ranging from m/z 1000 to m/z 1300. This specific mass range enabled the detection and analysis of compounds falling within this range^31^.

#### Nuclear magnetic resonance (NMR) analysis

The methodology presented in this study relies on the detection of electromagnetic radiation within the radio frequency range of 4–600 MHz, specifically focusing on ^1^H-NMR spectroscopy. NMR spectroscopy is extensively utilized for structural analysis and purity assessment due to its ability to provide detailed information. In this particular investigation, proton NMR (1H-NMR) spectra were acquired at a temperature of 27 °C using a Bruker AV-500 spectrometer from Germany operating at a frequency of 500.13 MHz. To prepare the samples for analysis, 10 mg of the sample was dissolved in 0.7 mL of deuterium oxide (D_2_O). The chemical shifts in the spectra were referenced using DSS (2,2-dimethyl-2-silapentane-5-sulfonate), a reliable reference compound widely employed in NMR spectroscopy^32^.

#### FT-IR spectral analysis

To determine the prominent functional groups present in both purified CyA and standard CyA, FT-IR spectra were acquired using an FT-IR spectrophotometer (Agilent Cary 660 series with a DTGS detector). The spectra were recorded across a scanning range of 400–4000 cm^−1^, with 32 scans performed for each sample. Prior to FT-IR analysis, the samples were dispersed in KBr pellets. This preparation method involves blending a small quantity of the sample with powdered KBr and applying pressure to form a transparent pellet. These pellets facilitate uniform and effective analysis of the samples using FT-IR spectroscopy, enabling the identification and characterization of the major functional groups present in the CyA samples^33^.

#### Raman spectroscopy

For Raman spectroscopy, a Takram P50C0R10 device was employed, which operates within the Raman scattering (RS) range of 100–4600 cm^−1^. In contrast to FT-IR spectroscopy, Raman spectroscopy employs laser light to investigate molecular vibrations, offering valuable insights into the structure and molecular bonds of purified CyA. By analyzing the reflected laser light, Raman spectroscopy enables the identification and characterization of molecular vibrations within the sample. This technique provides information about the specific bonds and functional groups present in purified CyA, thereby assisting in the determination of its molecular structure^34^.

#### X-ray diffraction (XRD)

Indeed, X-ray diffraction is a powerful technique that enables the determination of the crystal structure and arrangement of molecules within a sample. By analyzing the diffraction pattern resulting from the interaction of X-rays with the crystal lattice, valuable insights into the crystalline characteristics of both purified CyA and standard CyA can be obtained. In this study, an X-ray diffractometer (HR-XRD, Smart-LAB, Rigako Corporation, Tokyo, Japan) equipped with a rotating Cu anode was used to analyze the crystalline characteristics. The XRD scans were conducted over a range of diffraction angles (2θ) from 2.0° to 50°. A scanning rate of 6°/min was employed, and the measurements were performed at ambient temperature. This X-ray diffraction analysis provides detailed information about the crystal structure and arrangement of molecules in both purified CyA and standard CyA^35^.

#### Differential scanning calorimetry (DSC)

The physical state of CyA was examined using Differential Scanning Calorimetry (DSC) with a Pyris 1 instrument from Perkin-Elmer Corp., USA. During this analysis, the CyA samples were enclosed in sealed aluminum pans. The DSC measurements involved subjecting the samples to a temperature range of 25–800 °C. The DSC instrument recorded the heat flow curve in relation to temperature, producing a DSC thermogram. This DSC analysis provides information about the thermal behavior and transitions of CyA, allowing for the determination of its physical state and any associated phase changes^35^.

#### Zeta potential and DLS measurement

The Zeta potential of purified and standard samples of CyA was measured by preparing a concentration of 0.1% of the samples at different pH levels using sodium phosphate buffers (10 mM). The pH values tested ranged from 2 to 6. The Zeta potential measurements were conducted using a Malvern Zeta sizer instrument from Malvern Instruments Ltd., located in Worcestershire, UK. The instrument utilized a high-sensitivity digital camera to record the movement of particles. By analyzing the particle movement and distribution, the Zeta potential of the CyA samples was determined. Zeta potential is a measure of the electrostatic charge on the surface of particles and provides insights into their stability and potential interactions. Furthermore, the hydrodynamic size of the nanoparticles present in the samples was measured using the dynamic light scattering (DLS) method with the same Malvern Zeta sizer instrument. DLS allows for the characterization of size distribution and average size of particles in a liquid suspension, enabling the assessment of the nanoparticles present in the CyA samples^36^.

### Statistical analysis

The obtained data from the various tests were analyzed using a factorial experiment with a completely random design. To ensure reliability, each test was repeated three times. The data were then summarized by calculating the mean and standard deviation. Multiple comparison means were conducted using the Duncan test at a significance level of 5%. For the statistical analysis, SPSS Statistics 22 software was utilized. This software is widely recognized and employed for data analysis and interpretation, providing a robust platform for conducting statistical analyses and drawing meaningful conclusions from the data.

## Results and discussion

### Investigating the effect of solvent extraction on CyA production efficiency during fermentation

During a 10-day microbial fermentation process, a culture medium was utilized. The initial separation of the target compound, CyA, was performed using a centrifuge. Subsequently, the quantification of CyA in the supernatant solution was carried out using HPLC. The measured concentration of CyA in the supernatant was determined to be 334 mg/L. Dairy sludge, which is a byproduct of dairy factories, shares similarities in composition with milk and contains various nutrients such as proteins, fats, carbohydrates, and minerals. This composition makes it a cost-effective fermentation environment for the production of fermented products^37^.

In the subsequent step of the process, ethyl acetate solvent was utilized for extraction, resulting in a measured CyA concentration of 456 mg/L in the sample. A comparison of the CyA levels before and after extraction revealed a 36% increase in the amount of the metabolite. The non-polar and hydrophobic nature of CyA can be attributed to the abundance of methyl groups present in its amino acid residues. This characteristic makes CyA more soluble in non-polar solvents, such as alcohols, compared to water. The solubility of CyA in water, ethanol, and methylene chloride is reported as 0.04, 10, and 100 mg/mL, respectively. This information demonstrates the higher solubility of CyA in non-polar solvents like methylene chloride, which explains the increased concentration observed after the extraction step using ethyl acetate^38^. The choice of extracting solvent affects the structural properties of CyA, leading to variations in its solubility. When a non-polar solvent is used, the intramolecular hydrogen bonds within the β-sheet structure of CyA remain intact. However, the use of a polar solvent disrupts the three-dimensional conformation of CyA^39^. CyA, produced by fungi, exists in two forms: freely available in the fermentation medium and sequestered within the fungal vacuole. To obtain a higher yield of CyA, it is necessary to release it from the fungal vacuole. This requires a solvent that can create pores in the cell wall, traverse the cell membrane, and enter the fungal vacuole. The cell wall's rigid structure poses the primary barrier to material transport. Therefore, overcoming this resistance is crucial for successful extraction. Additionally, the solvent must possess the capability to dissolve the solid phase of CyA present in the vacuole. CyA is linked to lipoproteins and lipolytic molecules through various chemical and physical bonds within the mycelium. Thus, the solvent must be capable of breaking these bonds. Finally, the combination of the solvent and CyA needs to traverse the hyphal cellular compartments, the hyphal surface, and the solid–liquid interface through mass transfer. Ultimately, the solvent-CyA mixture should enter the liquid phase to complete the extraction process^38,40^. The use of ethyl acetate as a solvent for extracting CyA has shown promising results in increasing the yield of CyA. This solvent has demonstrated its effectiveness in traversing the cell wall, reaching the site where CyA accumulates, and overcoming barriers to mass transfer. Ethyl acetate has been successfully utilized by Balaraman and Mathew^41^ as well as Sharmila et al.^42^ for the isolation of CyA. Indeed, Tanseer and Anjum^25^ employed *n*-butyl solvent for the extraction of CyA from the culture medium of *Aspergillus terreus* strain. Likewise, Shetty et al.^43^ utilized ethyl acetate solvent for the extraction of antibiotics produced by *Streptomyces parvulus*. These studies highlight the versatility of different solvents in extracting bioactive compounds from various microbial sources^43^.

### Purification

**Figure 1:**
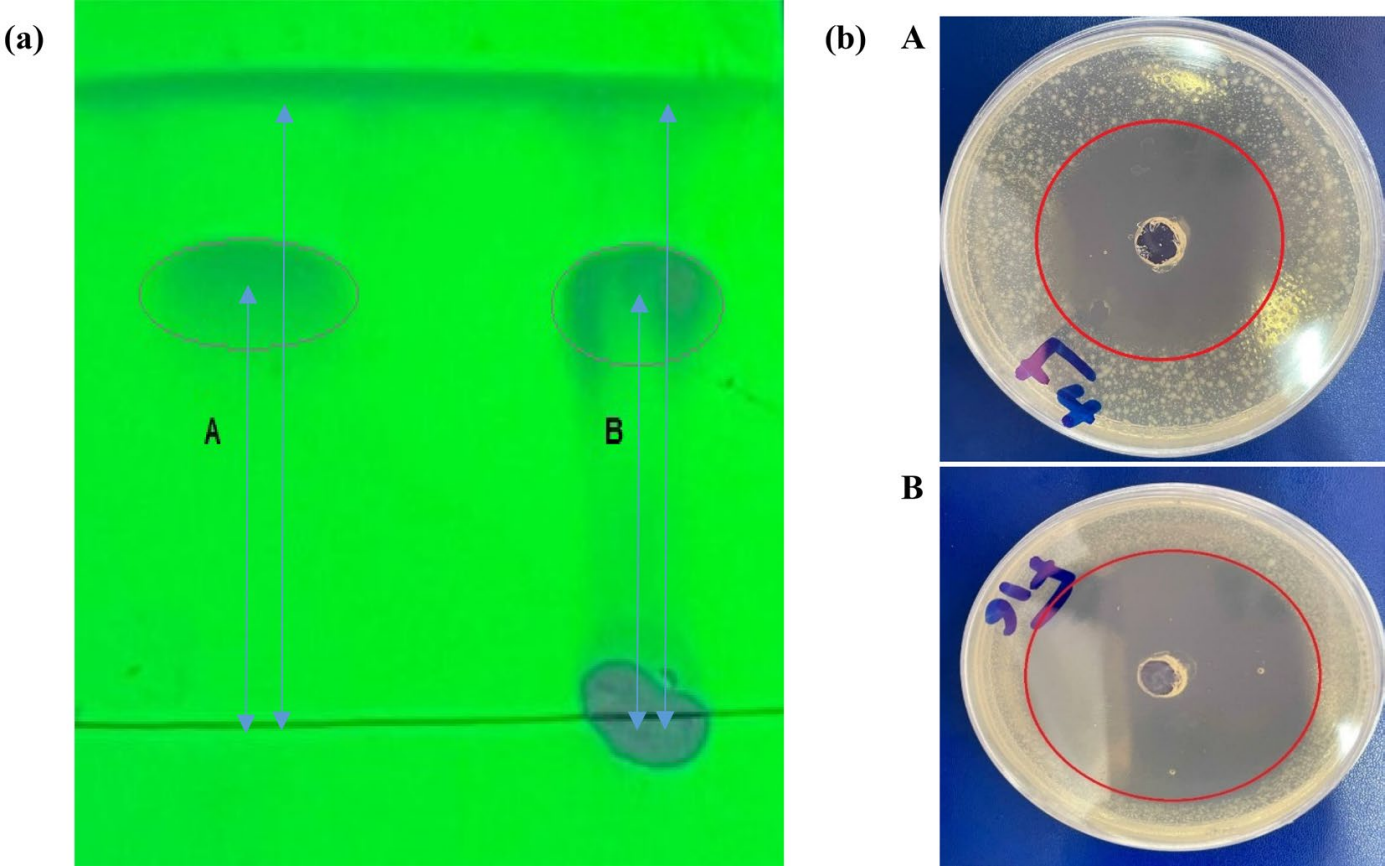
(**a**) TLC of purified (**A**), and standard (**B**) CyA samples at UV-254 nm. (**b**) The images of the plates obtained from the bioassay test related to the plate of the optimal sample (before extraction) (**A**), and the purified sample (**B**).

The purification process of CyA involved using FPLC packed with silica gel, and elution was carried out using a mixture of *n*-hexane and ethyl acetate in a 20:80 (v/v) ratio. This process resulted in obtaining a pure compound. To validate the samples obtained from the purification column, a TLC test was conducted. The sample that exhibited a line similar to that of the pure sample (Fig. 1a–A,B) was selected as the sample containing CyA. The CyA content of the pure sample was further measured using HPLC, and a value of 578 mg/L was determined. This represented a significant increase in the amount of CyA compared to the extracted and primary sample. The pure sample exhibited a 42% increase in CyA content compared to the initial sample before extraction, indicating the efficiency of the purification methods used. Consistent with previous research, TLC, column chromatography using silica gel, and HPLC were employed for the purification and measurement of CyA^42^. In addition to the purification process, the antimicrobial properties of the obtained sample were evaluated using a bioassay. The diameter of the clear zone formed around the fungal colony was examined before and after purification. It was observed that the diameter of the clear zone after purification was greater than that in the sample before purification (Fig. 1b–A,B). The size of the clear zone can be considered as an indicator of the concentration of CyA, as there is a direct relationship between the concentration and the size of the clear zone. This suggests that the purification process not only increased the concentration of CyA but also enhanced its antimicrobial activity, as evidenced by the larger clear zone observed in the bioassay. This indicates the potential of purified CyA as an effective antimicrobial agent^22^.

### Analysis of the structure of CyA

#### LC/MS/MS profiling

**Figure 2:**
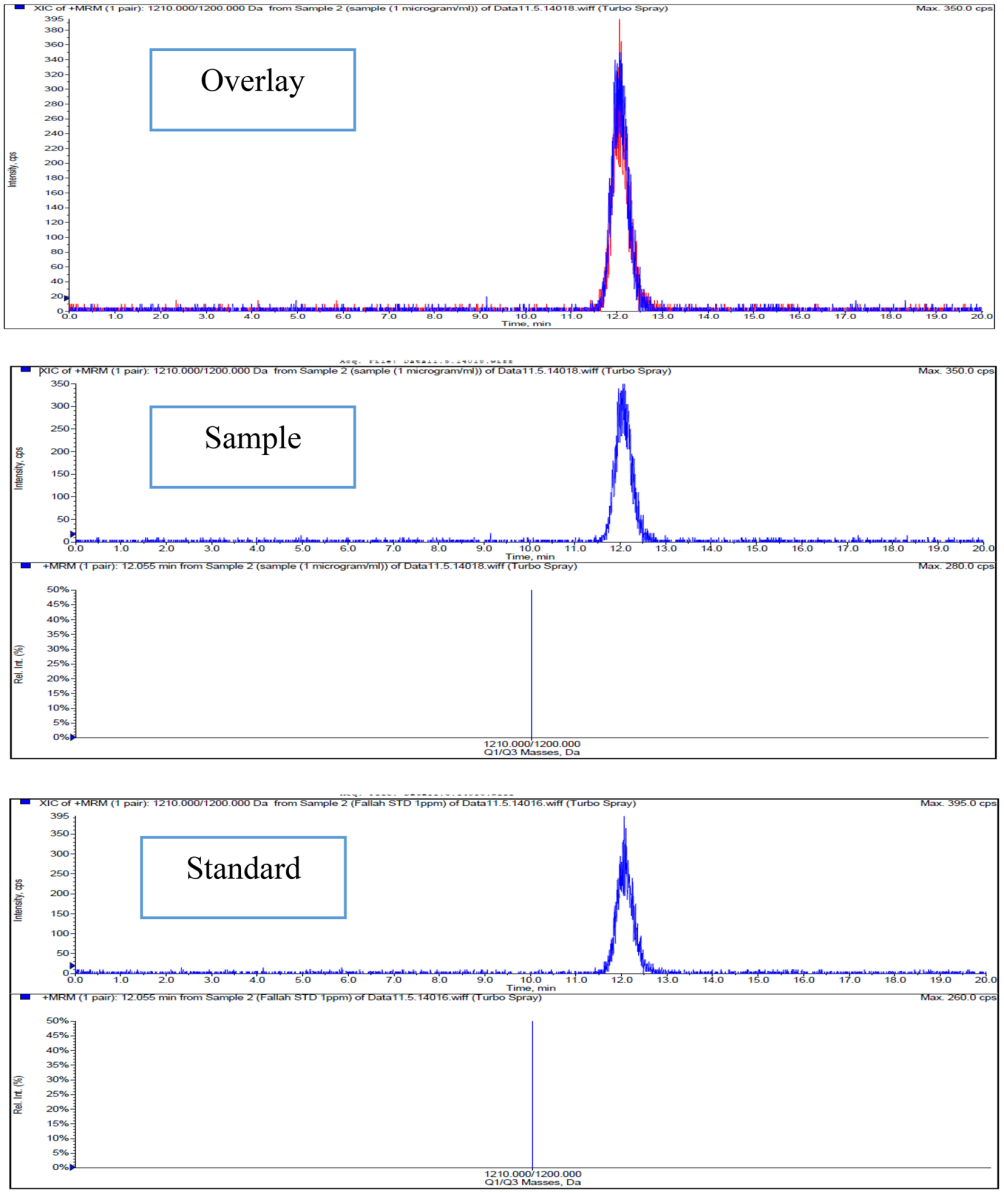
LC/MS/MS chromatograms of purified (up), and standard (down) CyA samples.

LC–MS/MS has indeed gained significant popularity and utility in clinical routine environments in recent years. This technique combines the separation power of liquid chromatography with the sensitivity and specificity of mass spectrometry. As a result, LC–MS/MS enables the precise identification and quantification of a wide range of analytes in complex biological samples. Its ability to handle diverse compounds and provide accurate measurements has made it an invaluable tool in various fields, including pharmacokinetic studies, drug monitoring, toxicology, and biomarker analysis. LC–MS/MS has revolutionized analytical chemistry and has become an indispensable technique in modern clinical research and diagnostics^44^. The LC–MS/MS method was utilized to identify metabolites from the purified fungal extract. The validation process involved analyzing accuracy and precision in three replicates. The standard analytical range of 9–1000 ng/mL was calculated for both the purified and standard CyA. Quantitative data was obtained using selected ion monitoring (SIM) of the hydrogen adduct of cyclosporine, with an m/z value of 1203, representing [M + H] + CyA. By comparing the LC–MS chromatogram of the standard CyA with the purified sample, the results confirmed the presence of CyA with an m/z value of 1203 [M + H]+, indicating successful identification. Based on the analysis of the chromatogram in terms of retention time for both the standard and purified samples, the retention time for CyA was found to be between 10 and 13 min (Fig. 2). This similarity in retention time between the two samples provides evidence for the presence of CyA. However, the standard CyA chromatogram appeared sharper than the purified sample chromatogram, indicating the presence of impurities in the sample. The purified sample was reported to have a purity value of 91.9%, as determined by LC/MS/MS. Furthermore, an investigation was conducted comparing the purified and standard CyA. It was observed that the peak area of the LC/MS chromatogram with an m/z value of 1203, corresponding to [M + H]+, was 2.1 times higher in the standard sample than in the purified one (Fig. 2). This difference in peak area suggests that the concentration of CyA in the purified sample is lower compared to the standard, indicating the need for further optimization of the purification process. Regarding the adducts ([M + H]+, [M + Na]+, [M + K]+), they refer to ions resulting from ion/molecule reactions, where the ion attaches itself to the molecule. These adducts can provide additional information about the molecular structure and behavior of the analyte.

The use of LC–MS/MS for assessing purity and detecting other metabolites has been widely reported in various research articles across different fields^45–48^. Abrol et al.^49^ conducted a study using LC–MS/MS to investigate the production of CyA by 11 mutant strains of *T. inflatum*. The LC–MS/MS profile of the crude fungal extract confirmed that both the wild type and mutant strains (MT1-3538, MT2-3538) were indeed more suitable for CyA production. Through analysis of the ion intensity profile, the researchers discovered that the mutant strain MT2-3538, when cultivated in an optimal media composition, exhibited a significant 16-fold increase in CyA efficiency compared to the other strains. This finding suggests that the specific genetic mutation in the MT2-3538 strain, along with the optimized growth conditions, had a substantial impact on enhancing the production of CyA^49^. Lam et al.^50^ conducted a study where they employed tandem mass spectrometry (MS/MS) to differentiate between various forms of CyA analogues. Specifically, their focus was on distinguishing between CyA and CycH (CyH), which are enantiomers, as well as isoCyA, a structural isomer of CyA and CycH. By utilizing the MS/MS technique, Lam et al. were able to fragment and analyze the molecular ions of these CyA analogues, enabling their differentiation based on their distinct fragmentation patterns. The MS/MS analysis provided valuable information about the mass-to-charge ratio (m/z) values of the fragmented ions, which proved instrumental in distinguishing between the different forms of the analogues. This study highlights the practicality of tandem mass spectrometry in accurately characterizing and differentiating closely related compounds such as CyA, CycH, and isoCyA^50^.

#### NMR

**Figure 3:**
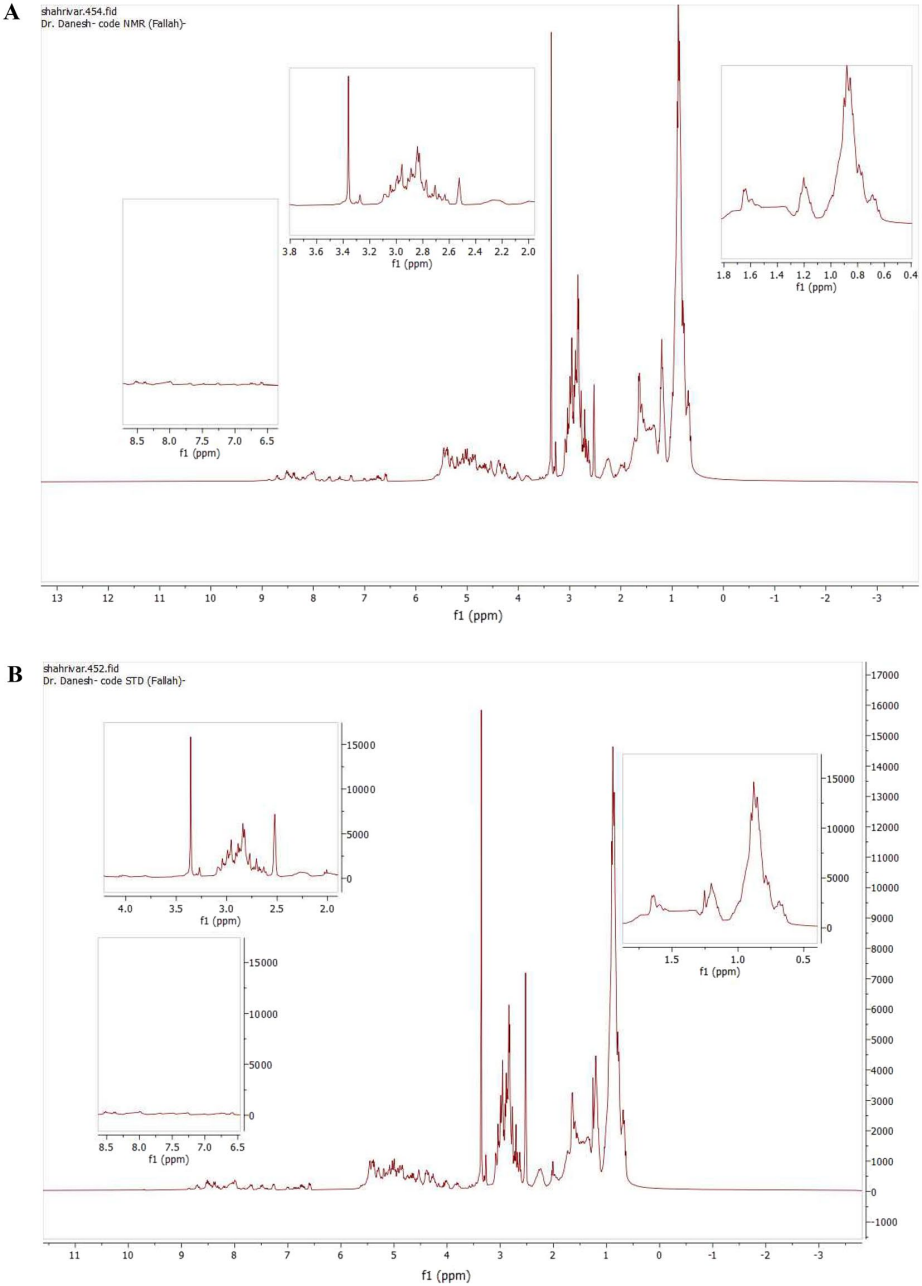
^1^H-NMR spectrum of purified (**A**), and standard (**B**) CyA samples.

H-NMR spectroscopy was utilized for the structural analysis and purity assessment of purified CyA. Figure 3 depicts the H-NMR spectra of both standard and purified CyA samples. CyA, with its intricate structure of cyclo-[-MeBmt1-Abu2-Sar3-MeLeu4-Val5-MeLeu6-Ala7-D-Ala8-MeLe9-MeLeu10-MeVal11], exhibits a highly complex proton spectrum in the high-field region. In both samples, the spectrum revealed the presence of four doublets in the 6–8 ppm range, corresponding to the amide protons. Additionally, approximately 23 protons, representing the protons of the 11 amino acids and olefinic protons, were observed in the 4–6 ppm range. Furthermore, the *N*-methyl protons were clearly distinguishable as seven distinct peaks within the 2.6–3.5 ppm range. Notably, the peaks' intensity was higher in the 0.5–3 and 7–8 ppm range, which aligns with the findings reported in previous H-NMR studies on CyA. The utilization of H-NMR spectroscopy provided valuable insights into the structural characteristics of purified CyA and facilitated the evaluation of its purity. The observed spectral features are consistent with the findings reported in previous studies, thereby validating the reliability and conformity of the obtained results^32,51,52^.

Figure 3 illustrates the chemical shifts corresponding to the protons of each amino acid segment in CyA. The spectra characteristics of the purified sample are displayed as follows: DAL8 HβHα (0.76` ppm), MLE9 Hβ2Hα (0.78 ppm), MLE10 Hβ2Hα (0.83 ppm), MLE6 Hβ2Hα (0.85 ppm), MLE4 Hβ2Hα (0.88 ppm), ALA7 HβHα (1.1 ppm), BMT1 HƞHζ (1.14 ppm), ABU2 HβHα (1.19 ppm), BMT1 Hδα2Hε (2.5 ppm), MLE9 Hβ1Hα (2.59 ppm), VAL11 HβHα (2.7 ppm), MLE4 Hβ1Hα (2.77 ppm), MLE6 Hβ1Hα (2.84 ppm), MLE10 Hβ1Hα (2.95 ppm), VAL5 HβHα (2.99 ppm), BMT1 HδβHε (3.05 ppm), BMT1 HβHα (3.35 ppm), VAL5 HNHα (6.5 ppm), DAL8 HNHα (6.67 ppm), ALA5 HNHα (8.4 ppm), ABU2 HNHα (8.5 ppm). The Schiff chemical of standard sample was DAL8 HβHα (0.76 ppm), MLE9 Hβ2Hα (0.78 ppm), MLE10 Hβ2Hα (0.83 ppm), MLE6 Hβ2Hα (0.85 ppm), MLE4 Hβ2Hα (0.88 ppm), ALA7 HβHα (1.1 ppm), BMT1 HƞHζ (1.14 ppm), ABU2 HβHα (1.20 ppm), BMT1 Hδα2Hε (2.52 ppm), MLE9 Hβ1Hα (2.62 ppm), VAL11 HβHα (2.7 ppm), MLE4 Hβ1Hα (2.8 ppm), MLE6 Hβ1Hα (2.83 ppm), MLE10 Hβ1Hα (2.92 ppm), VAL5 HβHα (2.98 ppm), BMT1 HδβHε (3.04 ppm), BMT1 HβHα (3.35 ppm), VAL5 HNHα (6.58 ppm), DAL8 HNHα (6.67 ppm), ALA5 HNHα (8.38 ppm), ABU2 HNHα (8.5 ppm)^52^. These values are in accordance with those found by Sinnaeve et al.^53^.

The comparison of H-NMR spectra between the standard and purified samples indicates that the peaks in the standard sample exhibit sharper characteristics compared to those in the purified sample. This discrepancy can be attributed to the presence of impurities within the structure of the purified CyA extract. Similar observations have been reported by Price et al.^19^ and Ohta et al.^54^, and Gendron^55^ in their respective investigations of CyA structure using H-NMR. These studies corroborate the findings obtained in our own study, providing further validation of the consistency and reliability of the observed peaks.

#### FT-IR and Raman spectroscopy

**Figure 4:**
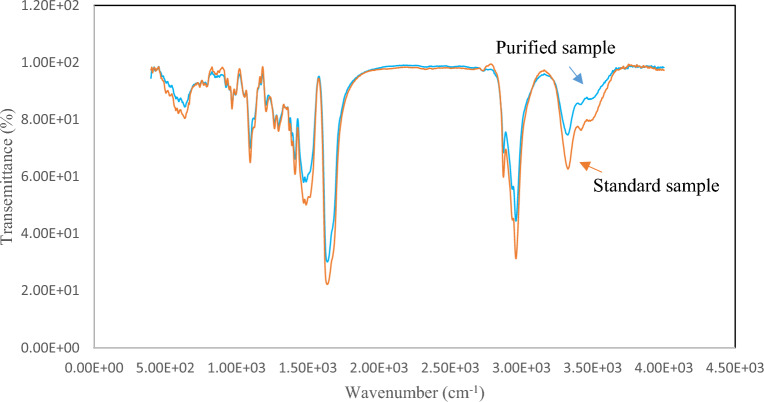
The FT-IR spectra of purified, and standard CyA samples.

To identify and detect the functional groups present in the structure of the purified CyA and compare them with the standard sample, we utilized FT-IR analysis and Raman spectroscopy. The results obtained from the FT-IR analysis, as depicted in Fig. 4, offer valuable insights into the functional groups present in the purified CyA sample.

In the structure of CyA, which consists of 11 different amino acids, there are numerous carboxyl and amine groups. During the FT-IR analysis, distinctive peaks corresponding to specific vibrational modes were observed in both the standard and purified samples. The N–H stretching and N–H bending vibrations, associated with the amine groups, display characteristic peaks in the broad absorption region of 3100–3500 cm^−1^ and 1410–1500 cm^−1^, respectively, in both samples. In the purified sample, specific peaks at 1206, 1266, and 1636 cm^−1^ indicate the C–O stretching vibrations of carboxyl groups, C–N stretching vibrations of amine groups, and C=O stretching vibrations of amide groups, respectively. Furthermore, the range of 2870–2960 cm^−1^ exhibits peaks that are characteristic of C–H stretching vibrations. Notably, in the purified sample chromatogram, distinct peaks at 791 and 1470 cm^−1^ can be attributed to the stretching and bending vibrations, respectively, of CH_2_ groups. These findings from the FT-IR analysis provide valuable insights into the presence and characteristics of various functional groups in the structure of purified CyA, allowing for a comparison with the standard sample^56^.

**Figure 5:**
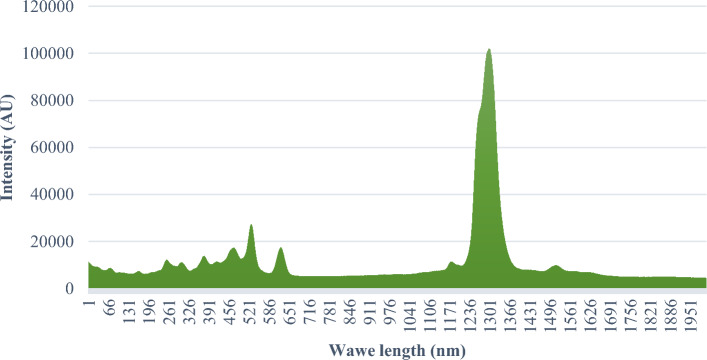
The complete Raman spectrum of purified sample.

The Raman spectrum of the purified sample can be observed in Fig. 5. In this spectrum, two bands are observed at 2944 and 2939 cm^−1^, which correspond to the stretching vibration of CH_2_ and CH_3_ groups, respectively. Additionally, a band at 1453 cm^−1^ is assigned as the bending vibration of N–H. A strong band is observed at 1665 cm^−1^, which is assigned as an in-plane vibration involving the C=O bond. Furthermore, rocking of NH_3_^+^ is observed at 1117 cm^−1^, while the antisymmetric bending vibration of CNH_2_ appears at 11,246 cm^−1^.

The bending vibrations of C–H were observed at 1325 and 1333 cm^−1^. The signal at 1315 cm^−1^ is related to stretching vibrations of C–O groups. Jenkins et al.^57^ employed Raman spectroscopy to examine the bonds and functional groups within the structure of CyA. Their analysis revealed the presence of amine, carbonyl, carboxyl, hydroxyl, and methyl groups in the structure of CyA^57^.

### Crystallinity and thermal analysis of the CyA

**Figure 6:**
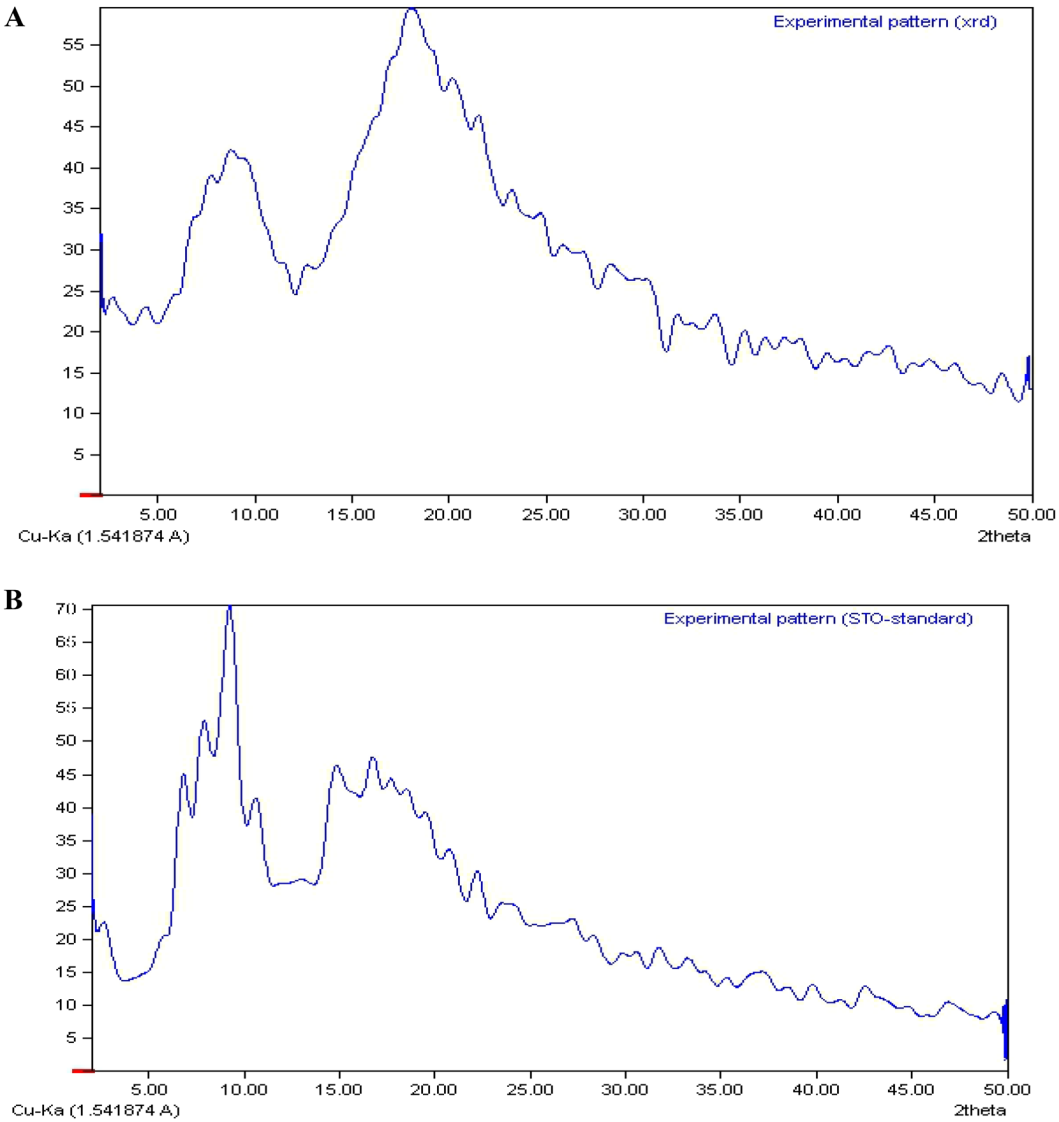
XRD pattern of purified (**A**), and standard (**B**) CyA samples.

The XRD test was conducted using Cu-Kα radiation at a voltage of 40 kV and a current of 40 mA. The scanning range of 2θ was set from 5° to 50°. In XRD analysis, the presence or absence of sharp diffraction peaks in the pattern is used to characterize a pharmaceutical powder as either crystalline or amorphous. As depicted in Fig. 6, both samples of CyA exhibited distinct diffraction peaks within the range of 5° to 45° (2θ), indicating a crystalline nature.

The XRD patterns obtained from both the lyophilized CyA (standard) and the purified CyA exhibited two broad peaks at 2θ = 9° and 19°, accompanied by characteristic narrow diffraction peaks. These findings indicate the presence of a semi-crystalline state in CyA, suggesting the absence of long-range three-dimensional order. The presence of broad peaks in the XRD patterns signifies a degree of disorder or amorphous nature in the samples. However, the presence of narrow diffraction peaks suggests the existence of localized ordering or crystalline domains within the overall semi-crystalline structure of CyA. The semi-crystalline nature of CyA, with the lack of long-range order, may be attributed to various factors such as molecular packing, intermolecular interactions, or the presence of impurities. Further investigations are required to fully comprehend the implications of this semi-crystalline state on the properties and behavior of CyA^58,59^. The intensity of the peak observed at 2θ = 9° in the purified sample is higher than the intensity of the peak at 2θ = 19°, which is in contrast to the standard sample. This difference in peak intensities suggests the presence of impurities in the structure of the purified sample, as well as the presence of water within its structure. These impurities and water content could contribute to the altered intensities of the peaks. This finding aligns with the results reported by Jain et al.^60^, where changes in peak intensities were observed after drying a sample using a freeze dryer. The presence of impurities and water in the purified sample may affect its crystalline structure, leading to variations in peak intensities in the XRD pattern. Further analysis and characterization are necessary to better understand the specific impact of these factors on the structural properties of the purified sample^60^. Two broad peaks at 2θ = 9° and 20°, and characteristic narrow diffraction peaks, suggesting the existence of the semi-crystalline state of CyA, which is in accordance with the findings of this study^61^. In the tetragonal crystal structure, CyA has been described as having a twisted antiparallel β-sheet conformation. Within the orthorhombic crystal structure, the β-OH group of the MeBmt 1 residue is capable of forming a bond with a structural water molecule present in the crystalline monohydrate. This water molecule, in turn, participates in intramolecular hydrogen bonding with the carboxyl group of the MeLeu10 residue (as depicted in Fig. 6). However, in the dried thermotropic liquid crystal conformation, it is suggested that the β-sheet, g-loop, type II β-turn, and MeBmt1 turn structures remain intact. This conformation differs from the three-dimensional crystal structures due to the loss of a g-turn structure resulting from the absence of a hydrogen bond between the amide of D-Ala 8 and the carbonyl oxygen of MeLeu6 58. In line with the findings of this study, the crystal structure of CyA has been confirmed by Sun et al.^35^ and Guada et al.^59^ through XRD analysis.

**Figure 7:**
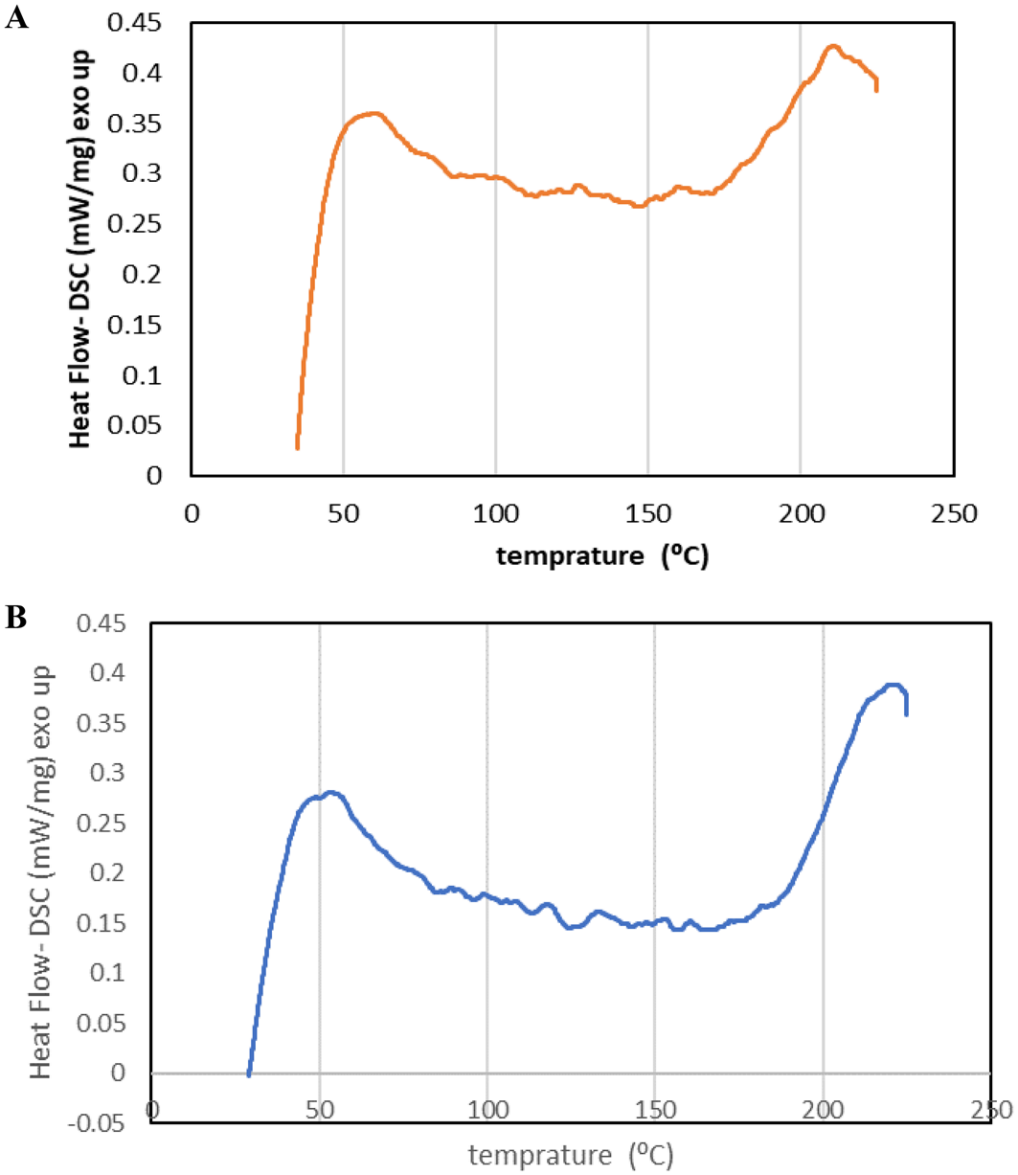
DSC curved of purified (**A**), and standard (**B**) CyA samples.

DSC is a powerful method used to identify the physical and chemical structure of materials and perform thermal analysis. It quantitatively measures energy changes and is employed to determine melting temperature, latent heat of melting, glass transition temperature (Tg), and crystallization temperature. Notably, the solid-to-liquid transition exhibited glass transition characteristics. The glass transition temperature can serve as a measure of macromolecular mobility, providing insights into the molecular mobility of CyA and changes in its semi-crystalline state^62^. Figure 7 represented the DSC curves of standard and purified CyA.

In this study, the glass transition temperatures of the purified and standard CyA samples were identified as 57–68 °C and 53–64 °C, respectively. The DSC thermogram revealed melting endothermic peaks ranging from 55 to 180 °C for the pure CyA sample and 70–200 °C for the standard CyA sample. Additionally, typical endothermic peaks indicating CyA crystallization temperature were observed at approximately 186–230 °C for the purified sample and 200–250 °C for the standard sample. The DSC curve analysis of CyA yielded consistent results, indicating that the glass transition temperature of pure CyA was found to be in the range of 51–55 °C^61^. The absence of variation in the lyophilized polymer and polymer of the device indicated an unchanged mobility from polymeric chains, and suggested the absence of detectable interactions between polymer and drug. In a study of Li et al.^63^, a temperature of 130 °C was reported for the crystallization of CyA. Sun et al.^35^ and Dubey et al.^64^ used DSC to investigate the thermal characteristics of CyA used in the nanofiber structure, which obtained similar results to this study. The study on CyA loading in lipid nanoparticles reported melting and crystallization temperatures of 140 °C and 200 °C, respectively, for pure CyA. These findings align with the results of the current study, indicating consistency in the temperature ranges for both melting and crystallization of CyA^59^. The DSC curve analysis of the purified CyA sample revealed a close resemblance to the standard sample. This similarity indicates that the thermal characteristics of these two samples are not significantly different from each other.

### Investigation of particle distribution and surface characteristics

**Figure 8:**
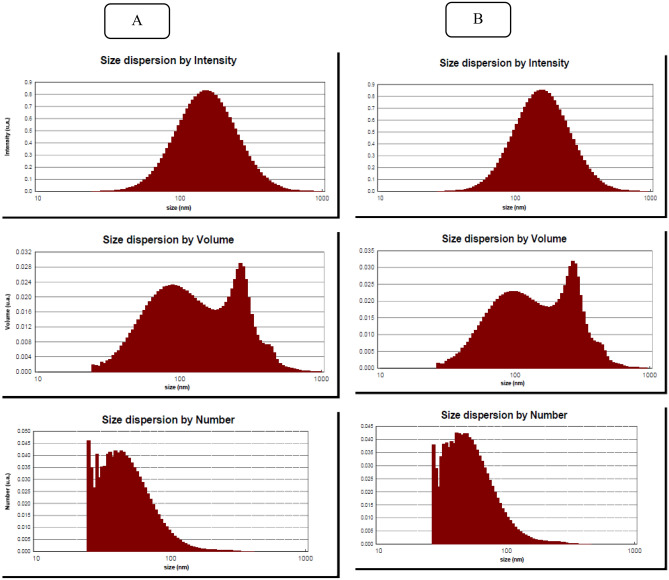
DLS curve related to purified (**A**), and standard (**B**) samples of CyA.

Particle size plays a crucial role in determining the solubility, spreadability, bioavailability, and effectiveness of materials. When particles are smaller, the ratio of surface area to volume increases. Consequently, this enhanced surface area provides more opportunities for substances to come into contact, react, and exert their effects, ultimately improving solubility and efficacy^65^. The analysis of the standard sample revealed an average particle diameter of 149.54 nm (intensity), 128.91 nm (volume), and 51.26 nm (number). In comparison, the refined sample exhibited an average particle size of 136.15 nm (intensity), 111.72 nm (volume), and 53.13 nm (number) (Fig. 8). These findings indicate a slight difference in particle size between the standard and purified samples. However, both samples displayed a uniform and similar particle size distribution. It is important to note that maintaining a narrow size distribution is crucial for preserving the functional characteristics and maximizing the effectiveness of particles. The extraction and purification processes employed in this research successfully generated particles with a uniform distribution, validating the accuracy of the downstream fermentation processes. The PDI (polydispersity index) values for both samples were low, indicating relatively homogeneous populations (less than 0.5). Furthermore, in a separate study, CyA-loaded micelles were reported to have a size of 137 nm and a PDI of less than 0.2, as per the results of this work^64^. Also, the particle size of 163 nm for encapsulated CyA was obtained^60^.

Zeta potential measurements were carried out to assess the stability of the electrostatic colloidal dispersion of the purified CyA particles and the standard sample. Both the pure and standard CyA particles were characterized for surface charge using a zeta meter. The zeta potential values obtained were − 25.8 ± 0.16 mV for the pure sample and − 23.63 ± 0.12 mV for the standard sample. A higher negative zeta potential indicates increased electrostatic repulsion between particles, which contributes to enhanced stability and favorable colloidal properties. In this case, the zeta potential of the purified CyA was not significantly different from that of the standard sample. However, there was a slight difference, which can be attributed to the lower purity of the purified sample. It is worth noting that the purified CyA sample did not exhibit any noticeable changes in appearance after being stored in an ambient atmosphere. This lack of change may be attributed to the high zeta potential, which could potentially prevent significant aggregation in the suspension^66^.

## Conclusion

CyA, a valuable and expensive fungal metabolite with medicinal importance, can now be produced economically through the use of low-cost fermentation medium, effective extraction techniques, and efficient purification methods. By employing dairy sludge as a nutrient source, CyA production reached 334 mg/L. Ethyl acetate extraction increased CyA yield by 36%. Purification using FPLC further enhanced CyA production to 578 mg/L, improving both purity and quantity. The purified sample exhibited a purity value of 91.9% as determined by LC/MS/MS analysis. H-NMR analysis confirmed the consistency of amino acid chemical shifts in both purified and standard samples. FT-IR and Raman spectroscopy analyses confirmed the presence of functional groups in CyA. Characterization studies using XRD and DSC compared the crystal structure, glass transition temperature, melting, and crystallization temperature of CyA in the purified sample to the standard sample. Both samples showed uniform particle size distribution, and there were no significant differences in zeta potential. Overall, the comprehensive analysis and comparison between the purified and standard samples revealed that impurities and side compounds in the purified sample were negligible. This study successfully optimized the production, extraction, and purification processes, resulting in a high-quality CyA product.

### Supplementary Information

Supplementary Information 1.

Supplementary Information 2.

Supplementary Information 3.

Supplementary Information 4.

Supplementary Information 5.

Supplementary Information 6.

Supplementary Information 7.

## Data Availability

All relevant data are in the manuscript.
