# Supplementary figures and images for: Bio-synthesis, purification and structural analysis of Cyclosporine-A produced by Tolypocladium inflatum with valorization of agro-industrial wastes
Source: Sci Rep. 2024 May 31;14:12540. doi: 10.1038/s41598-024-63110-y (PMC11143273; doi:10.1038/s41598-024-63110-y)

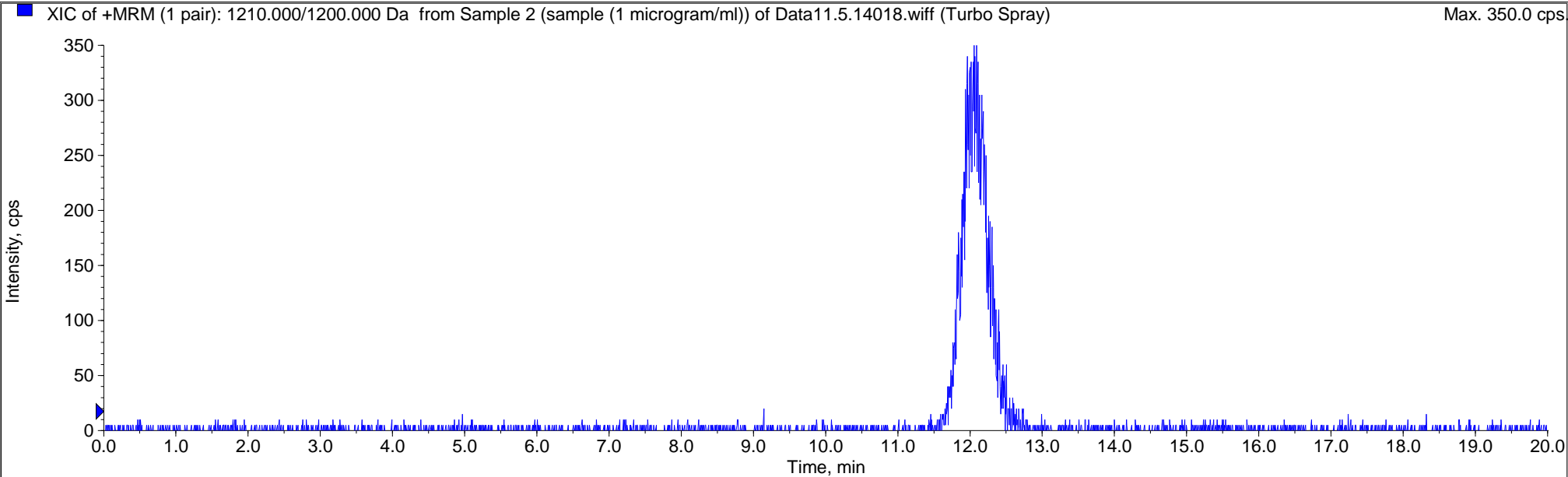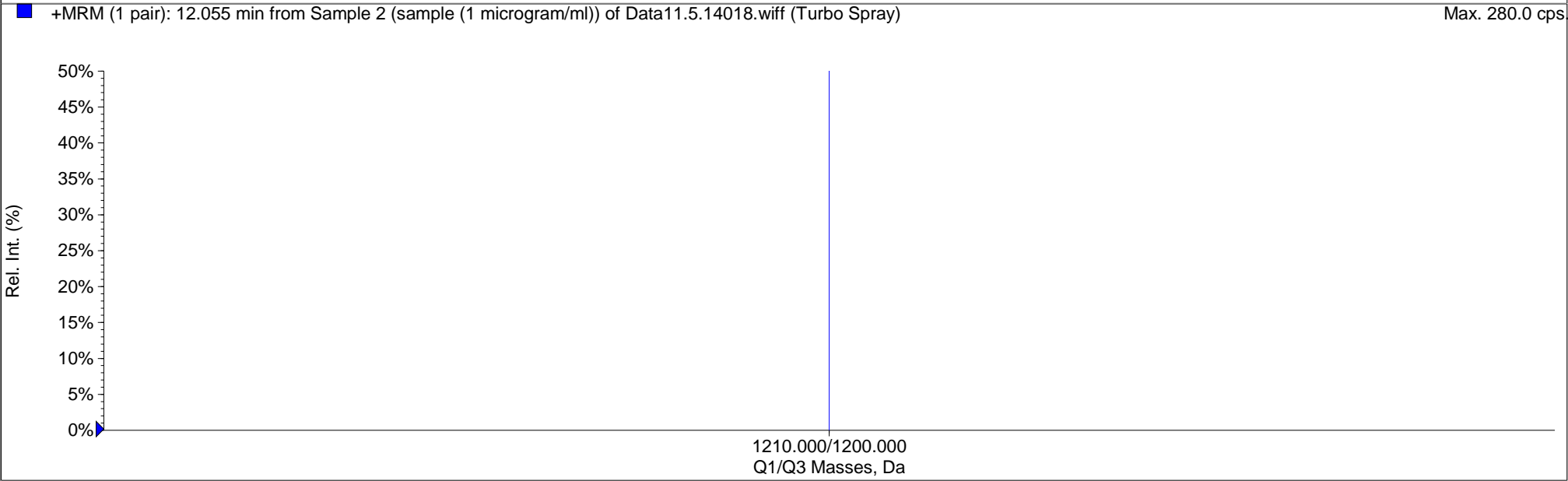

Supplement: Supplementary file 1 — Supplementary Information 1. [file 41598_2024_63110_MOESM1_ESM.pdf]

■ XIC of +MRM (1 pair): 1210.000/1200.000 Da from Sample 1 (STD 2) of Data23.5.14011.wiff (Turbo Spray), Smoothed

Max. 1618.5 cps

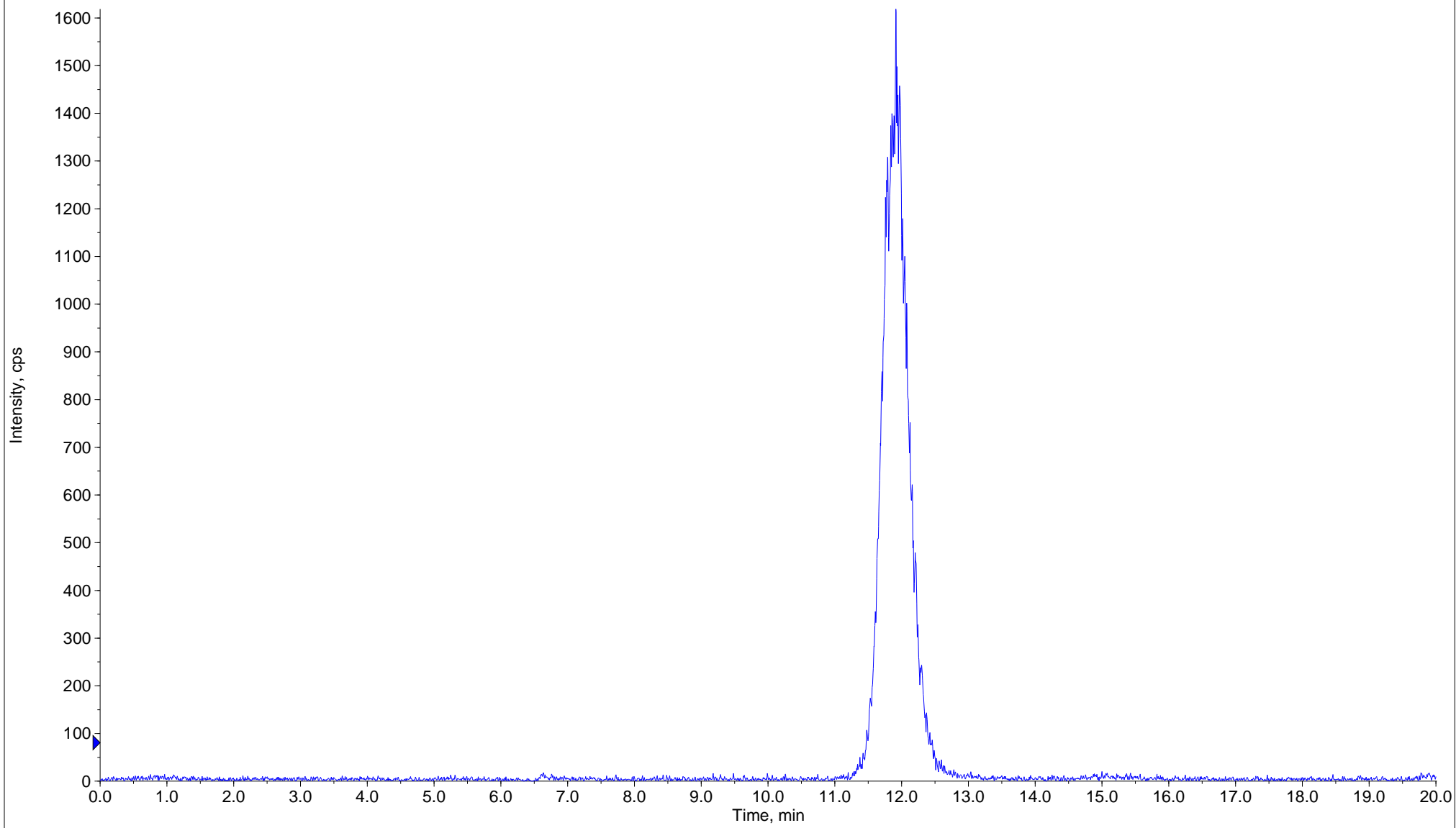

Supplement: Supplementary file 5 — Supplementary Information 5. [file 41598_2024_63110_MOESM5_ESM.pdf]

shahrivar.452.1.1r  
Dr. Danesh- code STD (Fallah)-

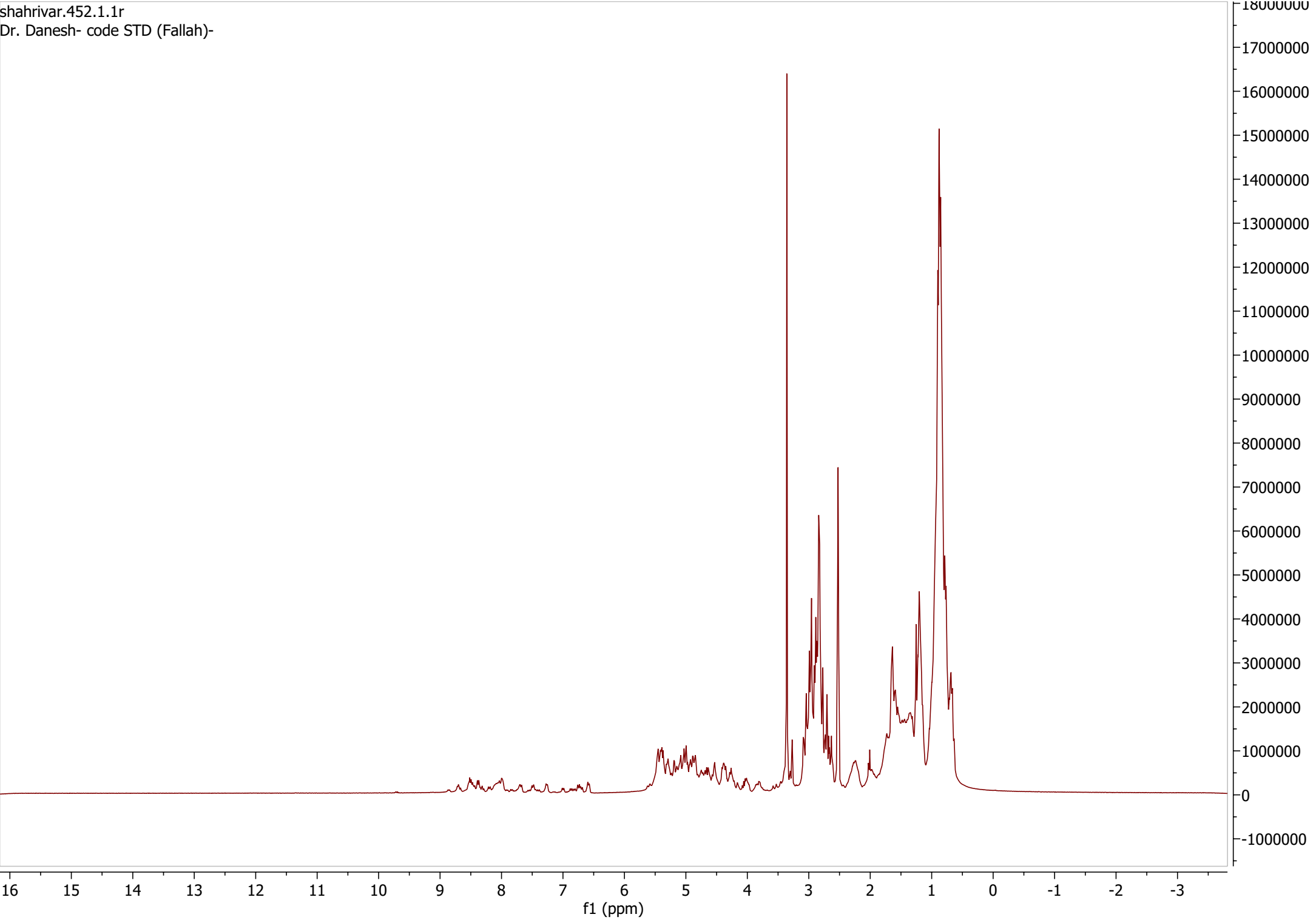

Supplement: Supplementary file 6 — Supplementary Information 6. [file 41598_2024_63110_MOESM6_ESM.pdf]

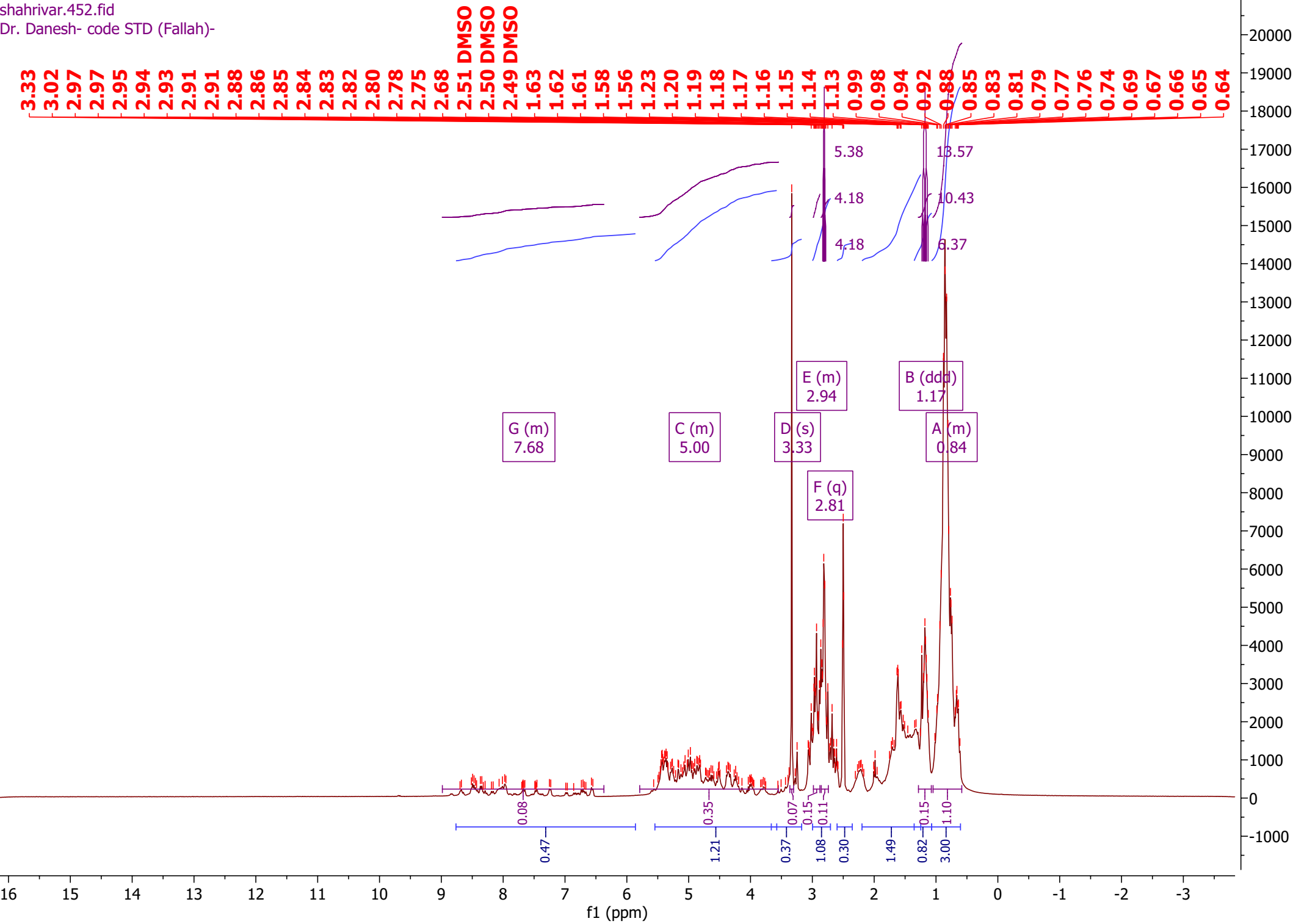

Supplement: Supplementary file 7 — Supplementary Information 7. [file 41598_2024_63110_MOESM7_ESM.pdf]
