# Supplementary material for: Bio-synthesis, purification and structural analysis of Cyclosporine-A produced by Tolypocladium inflatum with valorization of agro-industrial wastes
Source: Sci Rep. 2024 May 31;14:12540. doi: 10.1038/s41598-024-63110-y (PMC11143273; doi:10.1038/s41598-024-63110-y)

Printing Time: 10:55:53 AM
Printing Date: Wednesday, April 24, 2024

Scan Mode: Zero Width
Sample ID:
Batch Name: 11.5.1401.dab
Acq. File: Data11.5.14018.wiff

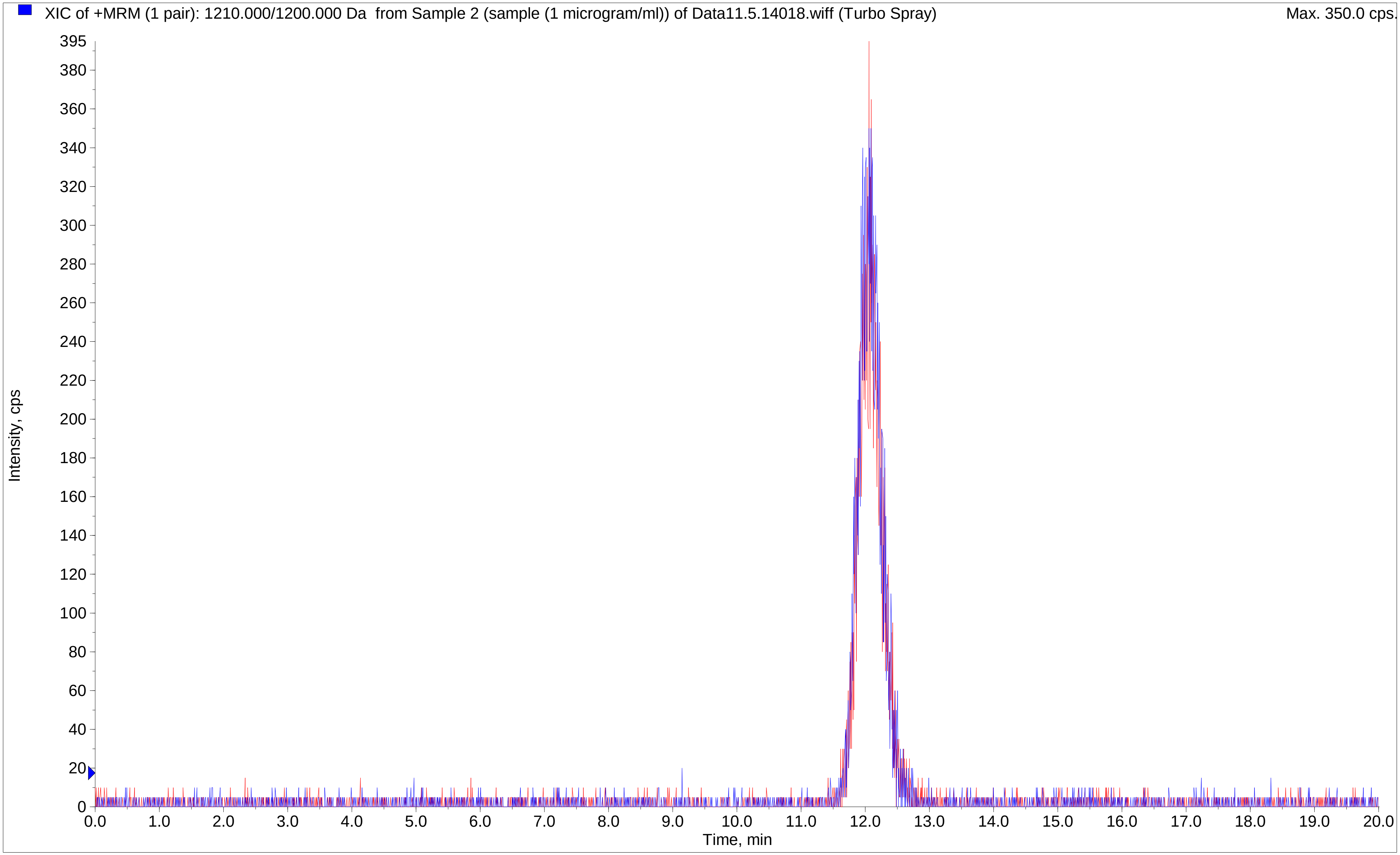

Supplement: Supplementary file 3 — Supplementary Information 3. [file 41598_2024_63110_MOESM3_ESM.pdf]
