# Supplementary material for: Bio-synthesis, purification and structural analysis of Cyclosporine-A produced by Tolypocladium inflatum with valorization of agro-industrial wastes
Source: Sci Rep. 2024 May 31;14:12540. doi: 10.1038/s41598-024-63110-y (PMC11143273; doi:10.1038/s41598-024-63110-y)
Supplement: Supplementary file 4 — Supplementary Information 4. [file 41598_2024_63110_MOESM4_ESM.pdf]

**Data File**	Data23.5.14015.wiff	**Result Table**	Fallah 23.5.1401.rdb
**Acquisition Date**	8/14/2022 2:29:56 PM	**Algorithm Used**	Analyst Classic
**Acquisition Method**	Fallah MRM2.dam	**Instrument Name**	3200 Q TRAP
**Project**	Buali\Buali subfolder 2017_01_31		

Sample Name: Sample 1 **Vial #:** 16

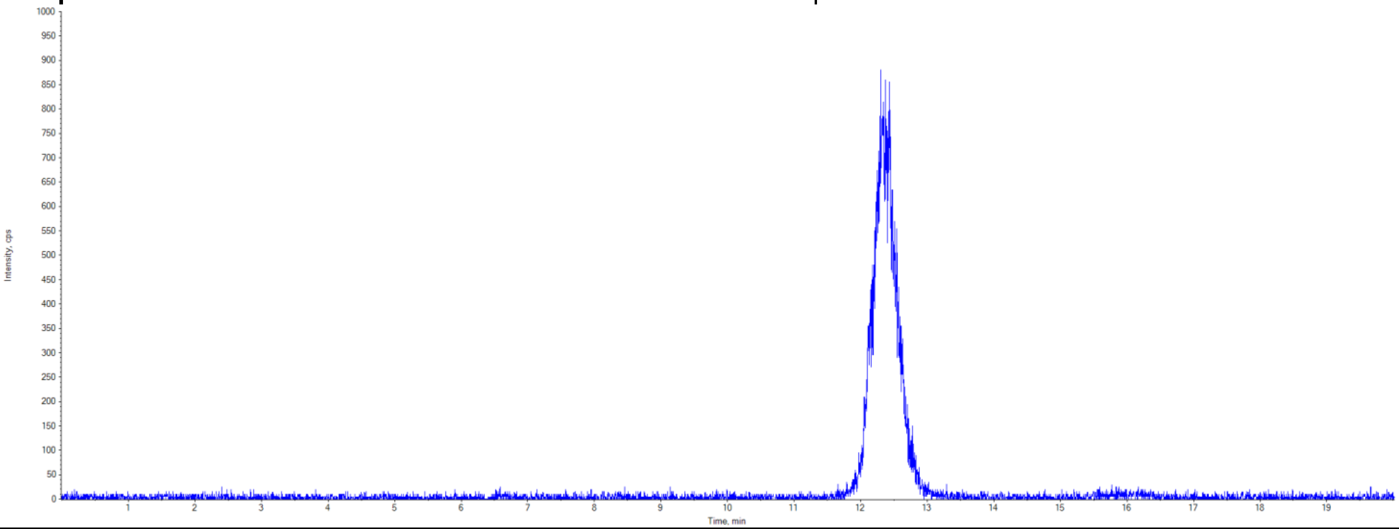

**Sample Name**	Sample 1	**Injection Vial**	16
**Data File**	Data23.5.14015.wiff	**Injection Volume**	20
**Acquisition Date**	8/14/2022 2:29:56 PM	**Algorithm Used**	Analyst Classic
**Acquisition Method**	Fallah MRM2.dam	**Sample Type**	Unknown
**Instrument Name**	3200 Q TRAP	**Result Table**	Fallah 23.5.1401.rdb
**Sample ID**	*No data for Sample ID*	**Dilution Factor**	1.00
**Sample Comment**	*No data for Sample Comment*	**Weight to Volume**	0.00

Approved By (Date and Initials):____.

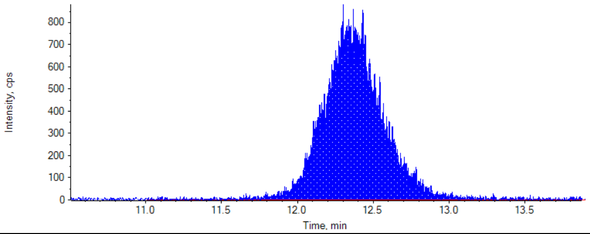

**Compound Name:**	1210.000 / 1200.000 **(**1210.000/1200.000 Da**)**
Expected RT:	12.2
Actual RT:	12.3
Equation:	y = 19.2 x + 2.86e+003 (r = 1.0000)
Area Counts:	2.05e+004
ISTD Area Counts:	N/A
Amount:	919. (ng/mL)
